# Results of NCCTG N0275 (Alliance) – a phase II trial evaluating resection followed by adjuvant radiation therapy for patients with desmoplastic melanoma

**DOI:** 10.1002/cam4.783

**Published:** 2016-07-01

**Authors:** William G. Rule, Jacob B. Allred, Barbara A. Pockaj, Svetomir N. Markovic, David J. DiCaudo, Lori A. Erickson, Richard L. Deming, Steven E. Schild

**Affiliations:** ^1^ Department of Radiation OncologyMayo ClinicPhoenixArizona; ^2^ Alliance Statistics and Data CenterMayo ClinicRochesterMinnesota; ^3^ Department of General SurgeryMayo ClinicPhoenixArizona; ^4^ Department of HematologyMayo ClinicRochesterMinnesota; ^5^ Department of DermatologyMayo ClinicRochesterMinnesota; ^6^ Department of Laboratory Medicine & PathologyMayo ClinicRochesterMinnesota; ^7^ Department of Radiation OncologyMercy Cancer CenterDes MoinesIowa

**Keywords:** Desmoplastic melanoma, radiotherapy, skin cancer

## Abstract

To examine, in a prospective fashion, the utilization and efficacy of adjuvant radiation therapy (RT) in patients with resected desmoplastic melanoma (DM). Adult patients with resected, margin‐negative, and nonmetastatic DM were eligible for this single‐arm prospective phase II study. Patients were to receive postoperative RT, 30 Gy in five fractions, to the operative bed with 2‐ to 3‐cm margins (depending on the tumor location). Nodal basin RT was not allowed. The primary study endpoint was the 2‐year local recurrence rate (LRR). Secondary endpoints included the incidence of regional and distant metastatic disease, progression‐free survival, overall survival (OS), and treatment‐related toxicity. Twenty patients with a single de novo DM lesion meeting trial eligibility criteria were enrolled and treated. The 2‐year LRR was 10%, with two patients demonstrating a LR within 2 years of completion of protocol therapy. No regional or distant failures occurred. OS at 2 and 5 years was 95 and 77%, respectively. There were no grade 3 or higher acute or late adverse events that were related to the protocol therapy. Adjuvant RT after wide local excision (WLE) for DM is efficacious and well tolerated. It should be considered for DM patients after margin‐negative WLE. Additional study is needed to further refine low‐risk patient populations that can potentially have adjuvant RT omitted as part of the treatment plan.

## Introduction

Desmoplastic melanoma (DM) is a rare variant of malignant melanoma that was originally described by Conley et al. 1. It is more common in males and the most common sites of involvement are sun‐exposed skin surfaces of the head and neck, followed by the extremities and trunk 2, 3, 4. These lesions can often be difficult to differentiate from other benign and malignant neoplasms, potentially leading to inaccurate diagnosis and inadequate treatment 5, 6. Historical local recurrence rates (LRRs) for patients with DM are in the range of 20–50% 2, 3, 4, 5, 7, 8, though recent series have suggested potentially lower rates in selected patients (negative margins, non‐head and neck location, lack of perineural invasion, decreasing Breslow depth, etc.) 9, 10, 11, 12. This is in contrast to patients with other cutaneous melanomas who have LRRs of 1–13%, depending on tumor depth and the presence or absence of ulceration 13, 14, 15. Higher rates of recurrence in DM patients have generally been attributed to the failure to adequately excise clinically occult projections of tumor 5, though many other tumor‐ and treatment‐related factors may play a role in LRRs (size of surgical margins, extent of desmoplasia, Breslow thickness, Clark level, head and neck location, neurotropism, etc.)^12^.

Given the high incidence of LR with surgery alone, rare regional lymphatic involvement, and a propensity for pulmonary metastases, the clinical behavior of DM appears to be similar to soft tissue sarcomas rather than other cutaneous melanomas 7, 16, 17. With retrospective studies of adjuvant hypofractionated radiation therapy (RT) demonstrating a benefit in locoregional control in high‐risk non‐DM patients versus historical controls, as well as anecdotal and retrospective reports of good clinical results in high‐risk DM patients, a prospective phase II study was designed to determine if adjuvant hypofractionated RT would result in low LR rates in selected DM patients 9, 18, 19, 20, 21, 22, 23, 24, 25.

## Methods and Materials

### Eligibility and follow‐up

Adult patients with resected, nonmetastatic, margin‐negative, and pathologically proven DM ≥1 mm in depth, or locally recurrent DM were eligible for enrollment on this IRB‐approved protocol after providing informed consent. Locally recurrent DM was defined as a DM lesion found ≤2 cm from a prior excision scar. Central pathologic review was required prior to enrollment. Only DMs as the predominant histologic pattern were allowed. Melanoma with focally desmoplastic features as well as nondesmoplastic neurotropic melanoma and nondesmoplastic spindle cell melanomas were specifically excluded.

Surgical margins for trunk and proximal extremities were recommended to be ≥2 cm, with margins for head and neck and distal extremities recommended to be <2 cm if necessary to preserve function and cosmesis. Patients with prior RT to the same site, a nonhealing surgical wound, and/or evidence of metastatic disease were excluded. Any planned adjuvant systemic therapy had to be deferred until after the completion of RT. A CT of the chest to rule out thoracic metastatic disease was required within 2 weeks prior to registration. After completion of RT, follow‐up physical examinations as well as chest X‐rays were performed every 3 months in follow‐up years 0–2 and every 6 months in follow‐up years 3–5.

### RT planning and delivery

CT‐based treatment planning was required for all patients. The required prescription dose was 30 Gy in five fractions of 6 Gy, administered twice per week over approximately 2.5 weeks. The dose was prescribed with electrons to Dmax with a point at the center of the incision. A point on central axis at the center of the incision at depth equal to the thickness of the tumor on the pathology report was required to receive at least 90% of the prescribed dose. Bolus was used as necessary to achieve a surface dose of at least 90% of the prescribed dose. Critical structures (e.g., brain, optic structures, spinal cord, brachial plexus, lung) were strictly prohibited from receiving a dose of 24 Gy or more. For tumors located in the head and neck region with a depth of ≤4 mm, 2‐cm margins between the estimated tumor bed (incision) and the block edges were used. For head and neck tumors with a depth greater than 4 mm as well as tumors in other locations, 3‐cm margins were used. Nodal basin RT was not allowed. RT was required to begin within 8 weeks after surgical resection.

### Study endpoints and statistical considerations

The planned study sample size was 20 patients. The primary study endpoint was the 2‐year LRR. LR, which was required to be biopsy proven, was defined as a DM lesion recurring within the radiated field (as described above). A simple binomial proportion, along with its 95% confidence interval, was used to estimate the true LRR. Secondary endpoints included the incidence of regional and distant metastatic disease, progression‐free survival (PFS; with progression defined as the first sign of disease progression or death due to any cause), overall survival (OS; time to death due to any cause), as well as treatment‐related toxicity (utilizing Common Toxicity Criteria version 2.0). Follow‐up time was calculated from the date of surgical resection. The Kaplan–Meier method was used to create survival curves for LR, PFS, and OS.

Data collection and statistical analyses were conducted by the Alliance Statistics and Data Center. Data quality was ensured by review of data by the Alliance Statistics and Data Center and by the study chairperson following Alliance policies. All analyses were based on the study database frozen on 11/15/2014.

## Results

A total of 20 patients who met the trial eligibility criteria were accrued and treated at seven different sites between November 2003 and May 2009. All patients had a single de novo DM lesion. The median follow‐up for surviving patients was 52 months (range: 30–65 months). The median patient age was 68 years (range: 49–76 years). A total of 50% of patients (10) were male and 50% (10) were female. None of the patients had received prior systemic therapy or radiation therapy prior to enrollment. See Table 1 for patient characteristics.

**Table 1:** Patient and disease characteristics

	Total (*N* = 20)
Age (year)	
*N*	20
Median	68.0
Range	(49.0–76.0)
Gender	
F	10 (50%)
M	10 (50%)
Desmoplastic histology	
Yes	20 (100%)
Primary tumor site	
Head/neck	11 (55%)
Upper extremity	5 (25%)
Trunk	3 (15%)
Lower extremity	1 (5%)
Operative procedure	
Wide local excision (WLE) of primary only	5 (25%)
WLE+ sentinel lymph node biopsy	13 (65%)
WLE+ lymph node dissection	2 (10%)
Breslow depth infiltration (mm)	
Median	3.0
Range	(1.0–21.0)
Clark's level	
IV‐reticular dermis	11 (55.0%)
V‐subcutaneous fat	9 (45.0%)
Surgical margins	
Negative, size not specified	4 (20%)
Negative, <1 cm clear	4 (20%)
Negative, 1–1.9 cm clear	6 (30%)
Negative, ≥2 cm clear	6 (30%)
Prior systemic therapy	
No	20 (100%)
Prior limb perfusion	
No	20 (100%)
Prior radiation therapy	
No	20 (100%)

Eleven (55%) lesions were from the head and neck region, five (25%) were from the upper extremities, three (15%) were from the trunk, and one (5%) was from a lower extremity. All patients underwent a wide local excision (WLE) of their lesions. The median Breslow depth was 3.0 mm (range 1.0–21.0 mm). Eleven patients (55%) had Clark's level IV disease and nine patients (45%) had Clark's level V disease. All patients had negative surgical margins. Six patients (30%) had margins ≥2 cm, six had margins between 1.0 and 1.9 cm, four (20%) had margins <1 cm, and four had negative margins with widths that were not specified. Thirteen patients (65%) also underwent a sentinel lymph node biopsy, with a median number of two nodes removed (range: 0–7). None of the nodes were found to harbor disease. Two patients (10%) underwent planned lymph node dissections with no positive nodes found. No in‐transit metastatic lesions were reported, but this was only specifically commented on in pathology reports for 16 (80%) patients. Table 1 presents pertinent pathologic findings.

All patients received RT per the protocol guidelines except for one, who had a major deviation secondary to a lack of submission of required documentation in order to completely assess the delivery of RT per protocol guidelines. That patient did not have a recurrence. All RT was delivered via en face electron fields with a median electron energy of 9 MeV (range: 6–20 MeV). Each plan was reviewed by multiple reviewers (including Dr. Steven Schild and staff of the Radiological Physics Center (RPC)) to assess protocol compliance.

**Figure 1:**
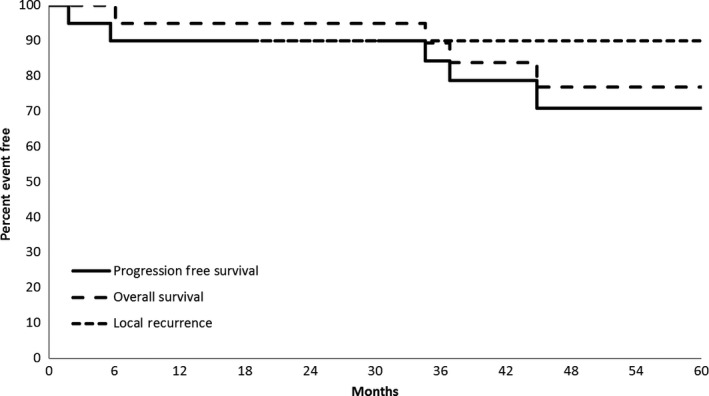
Local control, progression‐free survival, and overall survival.

The primary endpoint of 2‐year LRR was 10% (95% CI: 0–23.2%), with two out of 20 patients demonstrating a LR within 2 years of the completion of protocol therapy. Both patients had lesions of the head and neck region. The first patient was 72‐years old at the time of study entry. The negative margin width was <1 cm. A LR was noted at 5.7 months after study entry. The patient underwent surgical salvage. At last follow‐up, the patient was alive 5.1 years after study entry, with no overt evidence of DM recurrence. The second patient was 69‐years old at the time of study entry. The negative margin width was 1–1.9 cm. A LR was noted 5.1 months after study entry. The patient underwent surgical salvage. At last follow‐up, the patient was alive 3.8 years after study entry, with no overt evidence of DM recurrence. No further LRs have been noted in the entire cohort. No regional or distant failures have been noted. OS at 2 and 5 years was 95 and 77%, respectively. No patients died from DM. Figure 1 presents Kaplan–Meier curves for the distribution of LRR, PFS, and OS.

There were no grade 3 or higher acute or late adverse events that were related to the protocol therapy. The most common protocol therapy‐related toxicity was RT dermatitis. Fifteen patients (75%) developed grade 1 RT dermatitis and three patients (15%) developed grade 2 RT dermatitis. Table 2 presents treatment‐related toxicity/adverse events.

**Table 2:** Summary of related adverse events

Toxicity	Grade 1	Grade 2	Grade 3	Grade 4	Grade 5
Radiation dermatitis	15 (75%)	3 (15%)	0 (0%)	0 (0%)	0 (0%)
Pain due to radiation	3 (15%)	1 (5%)	0	0	0
Alopecia	2 (10%)	0 (0%)	0	0	0
Anorexia	1 (5%)	0 (0%)	0	0	0
Fatigue	0 (0%)	1 (5%)	0	0	0
Esophagitis	1 (5%)	0 (0%)	0	0	0

## Discussion

To the best of our knowledge, this is the first prospective trial specifically examining the role of adjuvant RT (electrons only) in patients with DM. Excellent local control results (90% at 5 years) were obtained. As a comparison, a retrospective study reported by Jaroszewski et al. 7 of 59 Mayo Clinic patients with DM evaluated the natural history of DM after surgical excision alone and found the overall LRR to be 39%. The majority of patients (61%) developed more than one LR. For the patients who developed a LR, the average depth of the original lesion was 10.6 mm, compared to 4.7 mm for the patients who did not develop a LR (*P* = 0.016). A LR was significantly more likely in patients who had unknown or positive margins (80%) than patients with negative margins (24%), *P* < 0.001. The LRR was noted to be much higher in DM patients with negative margins than in patients with other varieties of cutaneous melanomas. LR was a poor prognostic factor for the development of metastatic disease, with metastatic disease developing in 52% of patients with a LR versus 15% of patients without a LR (*P* = 0.006). Only one patient (2%) developed a regional lymph node metastasis. In the patients who developed distant metastatic disease, the most common sites were lung (81%), bone (25%), and brain (13%) 7. These findings are consistent with other reported series 5, 26, 27, 28.

More recently, Guadagnolo et al. 29 from MD Anderson Cancer Center retrospectively examined the role of adjuvant RT in DM. One hundred thirty consecutive patients with nonmetastatic DM presenting between 1985 and 2009 were evaluated. Fifty‐nine (45%) patients underwent WLE alone and 71 patients (55%) underwent WLE and postoperative RT. The vast majority of patients had negative final resection margins (93%). Sixty‐eight of the 71 patients who received postoperative RT were treated with the same fractionation regimen that was utilized in our trial (30 Gy in five fractions). As in our study, the majority of their patients had primary lesions of the head and neck region. The rate of LR for the entire population was 17% at 5 years. It was 24% for the patients who underwent WLE alone compared to 7% for patients who underwent adjuvant RT, consistent with our series (LRR: 10% at 5 years). On Cox multivariate regression modeling, local control was significantly improved with the addition of postoperative RT (*P *=* *0.009). OS at 5 years was 69%, also similar to our rate of 77% 29.

Another recent publication from Strom et al. 15 at Moffitt Cancer Center examined the role of RT in patients with DM. A total of 277 patients with nonmetastatic DM were treated between 1989 and 2010. One hundred thirteen patients (41%) received adjuvant RT. In this series, the majority of patients receiving RT prior to 2005 were treated with 30 Gy in five fractions. The majority of patients receiving RT after 2005 were treated with conventional fractionation using fractions of 1.8–2.0 Gy to total doses of 59.4–68 Gy. Adjuvant RT was found to be independently associated with an improvement in local control on multivariate analysis (HR 0.15, CI: 0.06–0.39, *P *<* *0.001). Twenty‐eight patients out of 164 (17%) who did not receive RT were noted to have a LR compared to eight out of 113 patients (7%) who received adjuvant RT. Five‐year actuarial local control rates were 95% for patients who received adjuvant RT, compared to 76% for patients who did not receive RT. Thirty‐five (27%) patients had positive margins. Those who received adjuvant RT had a significantly lower rate of developing a LR than those who did not (14% vs. 54%, *P *=* *0.004). While RT did significantly reduce the risk of LR, the LR rate was higher than in patients with negative margins. This certainly argues for re‐resection whenever feasible/tolerable in order to achieve negative margins. Of importance, the authors did identify two low‐risk groups in their analysis who could potentially omit adjuvant RT: (1) patients with negative margins, a non‐head and neck tumor location, and a Breslow depth ≤4 mm; and (2) patients with negative margins, a Breslow depth ≤4 mm, and no perineural invasion 15. Other authors have also noted patient populations at low risk for a LR who could potentially omit adjuvant RT 9, 10, 12. In general, these are patients with trunk or extremity DMs who are able to undergo a WLE with ≥2.0 cm margins with a thorough histopathologic review documenting negative margins. Table 3 presents a summary of LRRs across several studies (all retrospective except for this study) over the past decade.

**Table 3:** Local recurrence rates across selected desmoplastic melanoma studies

Study	Year of publication	Treatment time span	Number of patients	LRR surgery alone	LRR surgery and RT	Comments
Current study		2003–2009	20	NA	10%^a^	Prospective series.
Strom et al. 15	2014	1989–2010	277	24%^b^	5%^b^	Identified a subset of patients who could potentially omit adjuvant RT.
Guadagnolo et al. 29	2014	1985–2009	130	24%^b^	7%^b^	
Chen et al. 9	2008	1996–2007	128	5.9%^c^	7.4%^c^	Patients receiving adjuvant RT had more advanced tumors and narrower margins of excision.
Foote et al. 25	2008	1997–2006	24	NA	9%^d^	Postsurgical cohort with a high risk of LR.
Arora et al. 10	2005	1997–2004	49	4%^c^	NA	Majority of patients with WLE margins ≥2 cm.Remainder of patients with WLE ≥1 cm.
Gyorki et al. 12	2003	1996–2001	24	4%^e^	NA	All patients with WLE margins ≥2 cm.

All studies except for this study are retrospective. LR, local recurrence; RT, radiotherapy; NA, not applicable; WLE, wide local excision.

a 2‐year rate (simple binomial proportion).

b 5‐year actuarial rate.

c Crude rates.

d 3‐year actuarial rate.

e 2‐year actuarial rate.

Regarding RT dose/fractionation, as utilized in our study, 30 Gy in five fractions (planned and prescribed specifically as described above) is a commonly used regimen. Conventionally fractionated regimens are reasonable, as are moderately hypofractionated regimens, such as the standard postoperative Trans‐Tasman Radiation Oncology Group (TROG) regimen of 48 Gy in 20 fractions. This particular regimen has been noted to be quite tolerable in patients at risk of lymphatic region relapse after a therapeutic lymphadenectomy 30. Additionally, it was found to significantly decrease the risk of nodal failure in these patients who had conventional melanoma histologies.

While randomized trials exploring RT utilization specifically for the uncommon DM subtype are unlikely, TROG is currently accruing to a randomized trial of postoperative RT after WLE for neurotropic melanoma of the head and neck region (TROG 08.09). While this trial is not specifically for DM, it very likely that DM patients will make up at least a portion of the planned accrual target of 100 patients. It is hoped that this trial can shed further light onto the value and role of RT utilization in DM patients.

Limitations of our trial include its small sample size (due mainly to the rarity of DM), lack of a control group, lack of nuanced toxicity data, as well as the lack of comprehensive pathologic risk factors for all patients (perineural invasion, lymphovascular space invasion, ulceration, etc.). That being said, given our prospective data, as well as several retrospective reports, adjuvant RT should be considered for all patients with DM after margin‐negative WLE 8, 15, 25, 29. As noted above, there are selected DM patients who can potentially have adjuvant RT omitted as part of their treatment plan, though additional study is needed to further refine and define these low‐risk patient populations.

## Conflict of Interest

None declared.
